# Collateral damage to marine and terrestrial ecosystems from Yankee whaling in the 19th century

**DOI:** 10.1002/ece3.2542

**Published:** 2016-10-19

**Authors:** Joshua Drew, Elora H. López, Lucy Gill, Mallory McKeon, Nathan Miller, Madeline Steinberg, Christa Shen, Loren McClenachan

**Affiliations:** ^1^ Department of Ecology, Evolution and Environmental BiologyColumbia UniversityNew YorkNYUSA; ^2^ Department of Vertebrate ZoologyAmerican Museum of Natural HistoryNew YorkNYUSA; ^3^ Department of Environmental ScienceBarnard CollegeColumbia UniversityNew YorkNYUSA; ^4^ Department of Environmental StudiesColby CollegeWatervilleMEUSA; ^5^ Present address: Hopkins Marine StationDepartment of BiologyStanford UniversityPacific GroveCAUSA; ^6^ Present address: Nichols School of the EnvironmentDuke UniversityDurhamNCUSA

**Keywords:** conservation biology, historical ecology, marine/terrestrial linkages, shifting baselines

## Abstract

Yankee whalers of the 19th century had major impacts on populations of large whales, but these leviathans were not the only taxa targeted. Here, we describe the “collateral damage,” the opportunistic or targeted taking of nongreat whale species by the American whaling industry. Using data from 5,064 records from 79 whaling logs occurring between 1840 and 1901, we show that Yankee whalers captured 5,255 animals across three large ocean basins from 32 different taxonomic categories, including a wide range of marine and terrestrial species. The taxa with the greatest number of individuals captured were walruses (*Odobenus rosmarus*), ducks (family Anatidae), and cod (*Gadus* sp.). By biomass, the most captured species were walruses, grampus (a poorly defined group within Odontoceti), and seals (family Otariidae). The whalers captured over 2.4 million kg of nongreat whale meat equaling approximately 34 kg of meat per ship per day at sea. The species and areas targeted shifted over time in response to overexploitation of whale populations, with likely intensive local impacts on terrestrial species associated with multiyear whaling camps. Our results show that the ecosystem impacts of whaling reverberated on both marine and coastal environments.

## 1 Introduction

During the 19th century, hundreds of vessels left from American ports in search of large whales, primarily sperm (*Physeter macrocephalus*), right (*Eubalaena* spp.), bowhead (*Balaena mysticetus*), humpback (*Megaptera novaeangliae*), gray (*Eschrichtius robustus*) (Smith et al., 2012), and to a lesser extent “blackfish” or Pilot whales (*Globicephala* spp. Best, 1987;). These voyages were commercial ventures during which whalers sought out whales as sources of oil and whalebone, and they were immensely successful, with over 100,000 large whales killed by American whalers during the 1800s during the so‐called American‐style Pelagic’ era (Best, 1987; Reeves & Smith, 2006; Townsend, 1935). In addition to the animals captured, technological and environmental limitations resulted in large numbers of whales that were harpooned but not landed, often dying in the process (Scarff, 2001) This exploitation had effects on the whales’ population structure that are still visible today (Alter, Rynes, & Palumbi, 2007; Mesnick et al., 2011; Monsarrat et al., 2016; Roman & Palumbi, 2003; Ruegg et al., 2013).

Whaling voyages lasted from several months to over 5 years and covered tens of thousands of kilometers (Table 1). Because crews were typically paid in proportion to the total value of the catch, there was economic incentive to not return until the vessels’ holds were full. Subsequently, their voyages covered immense areas of open ocean (Smith et al., 2012). Whaling voyages represent some of the earliest, and in some cases the only, sources of historical ecological knowledge about the pelagic habits of these highly migratory animals, and the details within whalers’ logbooks give insight into marine ecosystems in the 19th century (Clapham et al., 2004; Townsend, 1935). In this way, a careful reading of logbooks can highlight how human perceptions of whale abundances have shifted over time (Pauly, 1995).

**Table 1:** Data from logbooks of ships of the 19th Century American Whaling Fleet. (1846–1901) * represents a ship lost during the Whaling Disaster of 1871 (see text)

Logbook ID	Ship name	Year(s)	Home port	Departure date	Return date	Days at sea
ODHS 450	Adeline	1850–1851	New Bedford, MA	9/20/1850	10/2/1851	377
KWM 13	Alfred Gibbs	1851–1854	New Bedford, MA	11/13/1851	7/20/1854	980
ODHS 448A	Almira	1864–1868	New Bedford, MA	8/10/1864	11/1/1866	813
ODHS 417C	America 2nd	1850	New Bedford, MA	2/23/1850	3/16/1850	21
ODHS 417B	America 2nd	1849–1850	New Bedford, MA	11/24/1849	1/22/1850	49
ODHS 417A	America 2nd	1849–1849	New Bedford, MA	4/2/1849	9/21/1849	172
ODHS 417D	America 2nd	1850–1851	New Bedford, MA	9/15/1850	7/14/1851	302
ODHS 980A	Beluga	1894–1896	San Francisco, CA	3/20/1894	11/20/1896	976
ODHS 951A	Beluga	1897–1899	San Francisco, CA	3/30/1897	3/04/1899	704
ODHS 952A	Beluga	1900–1901	San Francisco, CA	4/08/1900	11/7/1901	578
KWM 370	Betsey Williams	1851–1854	Stonington, CT	7/24/1851	4/20/1854	1,001
ODHS 848	Betsey Williams	1851–1854	Stonington, CT	7/24/1851	4/21/1854	1,001
ODHS 609A	Bounding Billow	1881–1882	Edgartown, MA	8/16/1881	9/18/1882	398
ODHS 698	California	1849–1851	New Bedford, MA	8/15/1849	3/10/1851	572
KWM 37	California	1894–1895	San Francisco, CA	12/4/1894	11/7/1895	338
ODHS 608B	Charles W. Morgan	1878–1881	New Bedford, MA	7/17/1878	5/11/1881	1,029
KWM 51B	Cicero	1853–1856	New Bedford, MA	7/7/1853	4/14/1856	1,012
ODHS 18	Cicero	1860–1865	New Bedford, MA	10/9/1860	5/25/1865	1,689
ODHS 413	Cleone	1858–1862	New Bedford, MA	11/5/1858	8/4/1862	823
ODHS 414	Cleone	1864–1868	New Bedford, MA	5/21/1864	6/14/1868	1,485
KWM 55	Congress	1864–1867	New Bedford, MA	5/31/1864	5/13/1867	1,077
ODHS 515	Daniel Webster	1848–1852	Nantucket, MA	5/19/1848	5/18/1852	1,460
ODHS 436A	Eliza Adams	1846–1849	Fairhaven, MA	6/12/1846	4/25/1849	1,048
KWM 319A	Eliza Adams	1851–1854	New Bedford, MA	11/3/1851	9/23/1854	1,370
KWM 74	Eliza Adams	1863–1867	New Bedford, MA	10/20/1863	4/22/1867	1,280
ODHS 995	Eliza F. Mason	1853–1857	New Bedford, MA	12/2/1853	4/10/1857	1,225
ODHS 609B	Fleetwing	1882–1883	San Francisco, CA	12/5/1882	11/4/1883	334
ODHS 385A	Fortune	1847–1850	New Bedford, MA	8/5/1847	6/6/1850	1,036
ODHS 385B	Fortune	1850–1854	New Bedford, MA	10/21/1850	5/18/1854	1,305
ODHS 994	Frances	1850–1852	New Bedford, MA	9/2/1850	10/24/1852	783
ODHS 669	Gay Head	1856–1860	New Bedford, MA	10/20/1856	8/28/1860	1,408
ODHS 948A	Grampus	1888	San Francisco, CA	2/11/1888	11/5/1888	268
ODHS 948B	Grampus	1889	San Francisco, CA	2/26/1889	11/12/1889	259
ODHS 6	Helen Snow	1871–1872	New Bedford, MA	10/17/1871	8/19/1872	307
ODHS 282	Henry Taber	1868–1871	New Bedford, MA	10/22/1868	9/14/1871*	1,057
ODHS 390	Hibernia	1866–1869	New Bedford, MA	11/21/1854	3/22/1856	487
KWM 105	Hudson	1855–1859	Fairhaven, MA	11/26/1855	4/25/1859	1,246
KWM 112	Islander	1865–1869	New Bedford, MA	11/12/1865	5/10/1869	1,275
ODHS 654A	John and Winthrop	1889–1890	San Francisco, CA	12/11/1889	11/7/1890	331
ODHS 769	John Wells	1869–1871	New Bedford, MA	11/9/1869	9/12/1871*	672
KWM 122A	Josephine	1856–1859	New Bedford, MA	7/15/1856	4/24/1859	1,013
KWM 122B	Josephine	1859–1862	New Bedford, MA	7/1/1859	7/1/1862	1,096
KWM 122C	Josephine	1863–1867	New Bedford, MA	4/14/1863	6/12/1867	1,520
KWM 130B	Louisa	1851–1853	New Bedford, MA	1/30/1851	1/21/1853	724
ODHS 608A	Louisa	1874–1878	New Bedford, MA	8/11/1874	5/3/1878	1,361
KWM 132	Lydia	1865–1869	New Bedford, MA	11/2/1865	5/1/1869	1,276
ODHS 392	Marcia	1857–1861	New Bedford, MA	8/25/1857	5/16/1861	1,360
ODHS 949	Mary D. Hume	1890–1892	San Francisco, CA	4/19/1890	11/29/1892	955
KWM 143	Mermaid	1896	San Francisco, CA	3/17/1896	11/10/1896	238
ODHS 395	Milo	1849–1851	New Bedford, MA	8/16/1849	7/20/1851	703
KWM 147	Milo	1863–1869	New Bedford, MA	11/26/1863	5/7/1869	1,989
ODHS 922	Moctezuma	1857–1861	New Bedford, MA	10/9/1857	4/11/1861	1,280
KWM 149	Mt. Vernon	1849–1852	New Bedford, MA	9/5/1849	5/18/1852	986
ODHS 614	Nassau	1850–1853	New Bedford, MA	8/5/1850	5/22/1853	1,021
ODHS 272	Navarch	1897	San Francisco, CA	3/2/1897	10/14/1897	226
KWM 155	Navy	1859–1864	New Bedford, MA	8/10/1859	4/18/1864	1,713
ODHS 749	Navy	1859–1864	New Bedford, MA	8/10/1859	4/18/1864	1,734
KWM 156	Navy	1869–1871	New Bedford, MA	10/7/1869	9/14/1871*	707
ODHS 950	Newport	1892–1898	San Francisco, CA	6/1/1892	11/26/1898	2,369
ODHS 399	Niagra	1851–1854	Fairhaven, MA	10/9/1851	2/17/1854	862
ODHS 946	Nimrod	1857–1861	New Bedford, MA	4/1/1858	7/12/1861	1,198
ODHS 981	Orca	1897	San Francisco, CA	11/30/1897	9/22/1897	176
KWM 51A	Phillipe de la Noye	1852–1854	Fairhaven, MA	9/6/1852	9/28/1855	1,117
ODHS 939	Progress	1880–1881	San Francisco, CA	12/16/1880	5/28/1881	163
KWM 319B	Roman	1851–1855	New Bedford, MA	12/21/1851	9/1/1855	1,350
KWM 176	Roman II	1850–1854	New Bedford, MA	8/1/1850	5/11/1854	1,379
ODHS 654B	Rosario	1891	San Francisco, CA	3/24/1891	11/6/1891	227
KWM 178	Rousseau	1849–1853	New Bedford, MA	5/9/1849	6/3/1853	1,486
ODHS 284	Rousseau	1853–1857	New Bedford, MA	10/17/1853	7/3/1857	1,355
ODHS 436B	Saratoga	1849–1852	New Bedford, MA	9/5/1849	4/26/1852	962
KWM 180	Saratoga	1857–1858	New Bedford, MA	4/27/1857	12/12/1858	594
KWM 181	Saratoga	1858–1860	New Bedford, MA	12/13/1858	6/1/1860	536
KWM 319C	Sea	1854–1855	Warren, RI	11/22/1854	4/9/1855	138
ODHS 7	Seneca	1869–1871	New Bedford, MA	10/16/1869	9/14/1871*	698
ODHS 993	Splendid	1862–1867	Edgartown, MA	8/11/1862	4/11/1867	1,704
ODHS 654C	Stamboul	1891–1892	San Francisco, CA	11/26/1891	10/24/1892	333
KWM 130A	Stephania	1847–1850	New Bedford, MA	9/15/1847	10/22/1850	1,133
KWM 192	Trident	1869–1871	New Bedford, MA	11/16/1869	6/10/1871	571
ODHS 644	Young Phoenix	1885	San Francisco, CA	2/21/1885	11/10/1885	262

While large whales were the primary targets of the American fleet (the so‐called Yankee whalers), they were not the only species targeted during these voyages. Infamously, 79 American whaling vessels captured over 13,000 Galapagos tortoises between 1831 and 1868 to serve as fresh meat on long voyages (Townsend, 1925). Similarly, Bockstoce and Botkin (1982) estimated that Yankee whalers killed over 200,000 walruses between 1848 and 1914. Thus, the ecosystem impacts of the American whaling fleet were not limited to the reduction in biomass and fixed carbon in the system due to the removal of large whales.

The capture of great whales can be viewed as individual captains opportunistically supplementing both the ship's oil holds and their pantries. Fresh meat was difficult to obtain along these voyages, and the chance to add new meat was rarely passed over. This gustatory enthusiasm for fresh meat even made its way into the most apocryphal of Yankee whaling tales, Moby Dick (Chapter 65: The Whale as a Dish. Melville, 1851). During the long periods between capturing large whales, other species would have provided the whalers a welcome diversion from preserved food and also occasionally additional sources of valuable oil. In particular, as whales became depleted, multiyear expeditions to more distant locales became necessary, requiring that overwintering whalers obtain provisions locally. Additionally, some species, such as walruses, were captured to provide additional income, through rendering to produce oil and the collection of tusks (Fay, Kelly, & Sease, 1989).

To fully understand the historical ecology of the marine ecosystems, we must rely on the data provided by the whalers themselves. While the history, ecology, and fisheries impacts of the large whale hunt have been well‐documented elsewhere (Herman, 1979; Smith et al., 2012; Townsend, 1935), the diversity of the other species targeted as well as their spatial distribution has not been fully explored. Here, we describe and quantify the diverse array of organisms other than large whale species captured by the American whaling fleet during the latter half of the 19th century (ships leaving port 1847–1900). In doing so, we have two main hypotheses. First, that because these were economic voyages, the majority of the nongreat whale catch recorded will be of species with economic value and not simply food items. Second, because of localized resource exploitation and increases in technology over time, we will see shift toward targeting populations in increasingly remote areas or species that were inaccessible with technology readily available during the beginning of the study period.

## 2 Materials and methods

We collected data from 79 digitized logbooks from the New Bedford Whaling Museum (NBWM) that cover a total of 74 voyages during the years 1846 to 1901 (Table 1). Logbooks from this period are not common, and the collections at the NBWM represent the largest collection of these documents. We focused on the latter half of the 18th century as it was during this time that the American Whaling fleet moved almost exclusively offshore from New England and the industry shifted from baleen to oil. It was during this time that the Arctic grounds were opened and American whaling was in its “golden era” (Dolin, 2008).

For each vessel, we recorded the unique logbook ID name, years active, home port, dates of departure and arrival, number of days at sea, and overall whaling grounds targeted. Within each logbook, we compiled records of the presence of nonwhale species captured. Exact longitude and latitude of each point of capture were recorded when possible, but many of the specific locality data were incomplete due to a lack of location observations during the examined period. In those circumstances, longitude and latitude coordinates were extrapolated from known locations within 10 days before or after the examined date, whenever possible (Table S1).

To quantify the level of exploitation, we listed the organisms captured to the most specific taxonomic resolution possible. When archaic terms were used, we used metadata such as geographic range, physical descriptions, or logbook illustrations to help refine taxonomic assignment. We calculated both absolute numbers of organisms caught and estimated approximate biomass of the total catch based on recorded average adult weights (Bigelow & Schroeder, 2002; Delacour, 1954; Nowak, 1999; Rice, 1998), although we used modern size data, we do note that species such as Cod (*Gadus morhua*, Hutchings & Baum, 2005) and Polar Bears (*Ursus maritimus* Rode, Amstrup, & Regehr, 2010) have undergone a recent reduction in size, and thus, our findings represent a conservative estimate of biomass. For species with extreme sexual dimorphism, we averaged between sexes as logbooks did not frequently differentiate (Prieto et al., 2013). For the taxonomic designation “grampus,” we used the weight of Cuvier's Beaked Whale (*Ziphius cavirostrus*, but see discussion below for the taxonomy of grampus).

We searched the historical literature to determine which species were associated with market goods (e.g., furs, oil) to differentiate between species targeted solely for food from those targeted for both food and opportunistic income supplementation.

To test the second hypothesis, that the fishery expanded in space (as measured by days at sea), we used a Mann–Whitney–Wilcoxon test, to assess averaged numbers of days at sea and numbers of individuals caught binned into before and after the ending of the US civil war (voyages starting 1846–1864 and 1865–1900, respectively). We chose this time to partition the data because after the US Civil war, there was an increase in well trained, and armed, men entering the fishery (Bockstoce & Botkin, 1982). Additionally, we calculated the diversity nonincidental (>10 individuals of a single species taken by a single vessel) catches by decade and analyzed spatial changes in nonincidental catch over time, which we associated with known changes in the abundance and availability of whales. Lastly, we calculated the total amount of contributions made to the total catch by strictly aquatic, semiaquatic, and strictly terrestrial animals.

## 3 Results

We collected data from 79 logs of which 56 (73.68%) reported catches of nongreat whale targets. These logs record the capture of 5,255 individuals of 32 different taxonomic designations (Table S1). The species with the greatest number of individuals caught were walruses (*Odobenus rosmarus N* = 2,283), ducks (Anatidae *N* = 949), and cod (*Gadus* sp., *N* = 524, Table 2). The species with the most biomass caught were walruses, “grampus,” and “seals” (Table 2). Overall walruses accounted for ~95% of the recorded catch by weight, and 43.3% of the total number of recorded individuals. Together, these 74 vessels caught approximately 2,439,812 kg of nonlarge whale species over 71,064 days at sea, equaling roughly 32,970 kg per vessel per trip or 34.3 kg per day at sea.

**Table 2:** Summary of nongreat whale catches made by the 19th Century American Whaling Fleet (1846–1901)

Species	Number	Apx. average weight	Apx. total weight	Habitat	Nonfood products?	Marine	Terrestrial	Semiaquatic
Walrus	2,283	1,000	2,283,000	Semiaquatic	Yes			2,283,000
Duck	949	1.5	1,423.5	Semiaquatic	No			1,423.5
Codfish	524	35	18,340	Marine	No	18,340		
Deer	292	80	23,360	Terrestrial	Yes		23,360	
Grouse	215	0.6	129	Terrestrial	Yes		129	
Fish	200	1	200	Marine	No	200		
Ptarmigan	165	0.5	82.5	Terrestrial	No		82.5	
Rabbit	151	2	302	Terrestrial	Yes		302	
Seal	85	300	25,500	Semiaquatic	Yes			25,500
Porpoise	84	80	6,720	Marine	Yes	6,720		
Fox	78	6.8	530.4	Terrestrial	Yes		530.4	
White Fox	51	5	255	Terrestrial	Yes			
Common Murre	43	1	43	Semiaquatic	No			43
Turtle	31	140	4,340	Marine	No	4,340		
Polar Bear	17	400	6,800	Semiaquatic	Yes			6,800
Skipjack	15	10	150	Marine	No	150		
Sunfish	13	1,000	13,000	Marine	No	13,000		
Grampus	9	5,000	45,000	Marine	Yes	45,000		
Fur seal	8	100	800	Semiaquatic	Yes			800
Bear	7	500	3,500	Terrestrial	Yes		3,500	
Moose	7	400	2,800	Terrestrial	Yes		2,800	
Albacore	7	50	350	Marine	No	350		
Dolphin	5	175	875	Marine	Yes	875		
Shark	5	100	500	Marine	No	500		
Beaver	4	20	80	Terrestrial	Yes		80	
Brown Bear	3	500	1,500	Terrestrial	Yes		1,500	
Kangaroo	2	90	180	Terrestrial	No		180	
Goose	2	5	10	Terrestrial	No		10	
Chicken	2	1	2	Terrestrial	No		2	
Sea otter	1	35	35	Semiaquatic	Yes			35
Grouper	1	4	4	Marine	No	4		
Wild pigeon	1	1	1	Terrestrial	No		1	

**Figure 1:**
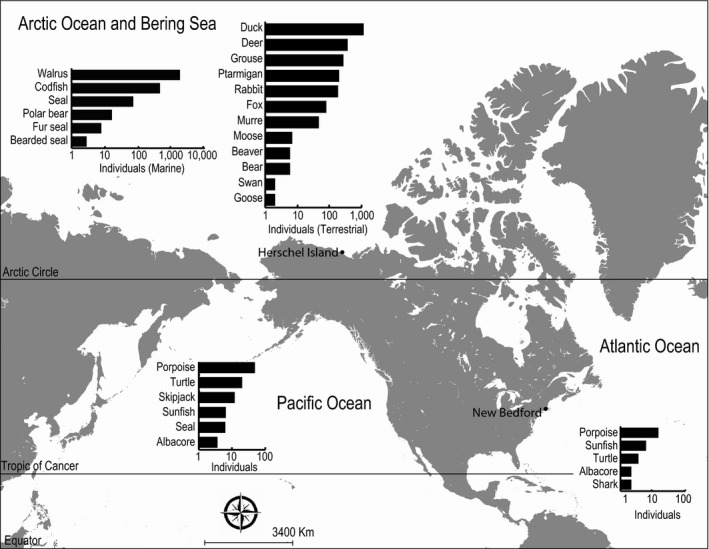
Marine and terrestrial species caught incidentally by Yankee whalers. Graphs represent number of individuals (log scale) taken on 74 voyages leaving from New Bedford, MA between 1846 and 1901 for each of three ocean basins. Individual animals in the Atlantic and Pacific Oceans were taken en route, while those in the Bering Sea and Arctic Ocean were taken while whaling or at whaling camps (e.g., Hershel Island)

There are strong spatial patterns of catch (Figure 1), with the majority of individuals and species targeted in the Arctic, where the whalers spent most of their time. Species targeted in the Atlantic and Pacific were primarily marine species, which reflects species taken as part of transit between New England and the Arctic whaling ground. The most commonly caught species in both the Atlantic and Pacific Oceans was porpoise, followed by turtle in the Pacific, and sunfish in the Atlantic. In the Arctic and Bering Seas, both marine and terrestrial species were taken in great quantities, reflecting the large amount of time spent in this region. Notably, the total number of terrestrial species taken from the Arctic exceeds the number of marine species, with popular game species like duck and deer representing the largest number of individuals taken.

The temporal patterns showed a heterogeneous pattern of exploitation. First, significantly more exploitation of nongreat whales took place after 1865 (4,826 of 5,064 recorded events, 95.3% W = 911, *p *< .01), which is rendered even more important after factoring in the shorter duration of voyages after 1865 (W = 325, *p *<< .001).

When we considered the targeted catches (>10 individuals of a single species taken by a single vessel; Table 3), we found strong spatial and temporal patterns in nonwhale catch that were associated with changes in the abundance of whales and the development of new technologies. In the early period (pre 1860s), whalers targeted beluga and other whales in the Chukchi Sea and Bering Sea. In this period, catches of nonwhale species represented low diversity in terms of both richness and evenness (Table 4). Indeed, walrus represented the only species caught nonincidentally in the 1860s and 1870s. By the 1890s, whales in this region were severely depleted, and new steam‐powered vessels allowed whalers to move into what is now the United States and Canadian Arctic to target bowhead whales. In response, associated collateral catch in this region increased in this decade (Table 3). As well, whaling voyages required overwinter stays to make trips profitable (Bockstoce, 1986). In response, the diversity of nonwhale catch increased (Table 4), reflecting a shift to subsistence hunting as whalers became reliant on local provisioning of locally abundant game species like ducks, deer, grouse, ptarmigan, and rabbit (Table 5).

**Table 3:** Nonincidental catch, or ten or more individuals of one species taken by a single ship. Here, we report only nonincidental catch that was associated with a known location

Species	Number	Year	Dates	Location	Ship Name
Turtle	10	1851	3 February	Halmahera (west Pacific)	Niagra
Duck	31	1851	12 July	Bering Sea (62.26N, 179.035 E)	Roman 2nd
Walrus	15	1859	12 August	Chukchi Sea: Cape Lisburne	Moctezuma
Walrus	14	1864	11 July	Chukchi Sea (68.00N, 171.47E)	Cicero
Walrus	26	1865	16–25 July	Chukchi Sea, 3 locations (69.29N, 163.29W; 69.19N)	Congress
Walrus	11	1867	2 July	Chukchi Sea (68.44N, 172.28E)	Hibernia
Walrus	212	1870	1 July–4 August	Bering Strait & Arctic Ocean (specific location unreported)	John Wells
Walrus	40	1870	2–8 July	Chukchi Sea, 3 locations (68.02N, 120.57W; 67.5N)	Henry Taber
Walrus	615	1870	2 July–4 August	Chukchi Sea, 4 locations (172.14; 67.20N; 57.19N; 70.09N)	Trident
Walrus	288	1870	4–31 July	Chukchi Sea, 2 locations (68.06N, 168.34W; 67.25N)	Seneca
Walrus	350	1870	17–31 July	Arctic, 5 locations (67.05N, 67.17N, 67.35N, 67.44N, 68.06N)	Navy
Walrus	240	1871	23 June–3 July	Bering Sea and Arctic Ocean, 3+ locations (Diomede, Cape Dezhnev, unreported)	Henry Taber
Walrus	197	1871	24 June–23 July	Bering Sea and Arctic Ocean, 3+ locations (Diomede, Western Arctic, unreported)	John Wells
Walrus	146	1871	16 June–15 July	Chukchi Sea, 6 locations (60.16N; 66.38N; 68.00N; 67.54N; 68.08Nm 170.29W; 67.41N)	Seneca
Walrus	23	1872	10 July	Bering Sea (65.32N, 170.37)	Helen Snow
Walrus	28	1885	10–11 May	Bering Sea (63.03N, 167.30W)	Young Phoenix
Common Murre	42	1888	10 June	Bering Sea (61.34N)	Grampus
Codfish	520	1889	13–16 April	Bering Sea, 3 locations (53.48N, 165.33E; 57.34N, 172.23E; 61.12N, 172.46E)	Grampus
Grouse	169	1891	24 March, 9 April	Eastern Arctic: Richard's Island	Mary D. Hume
Duck	134	1891	6–18 October	Gulf of Alaska: Orca Bay	Mary D. Hume
Grouse	15	1891	9 November	Gulf of Alaska: Orca Bay	Mary D. Hume
White fox	28	1891–1892	27 November–7 April	Gulf of Alaska: Orca Bay	Mary D. Hume
Deer	53	1892	9 May–3 June	Gulf of Alaska: Orca Bay	Mary D. Hume
Eider Duck	96	1893	2–6 November	Beaufort Sea: Herschel Island	Newport
Ptarmigan	119	1894	24 February	Beaufort Sea: Herschel Island	Newport
Deer	76	1894	21 April–7 June	Beaufort Sea: Herschel Island	Newport
Deer	37	1894	12 July	Gulf of Alaska: Perry Island	Newport
Duck	14	1894	30 July	Beaufort Sea: Russell Inlet	Newport
Duck	91	1894	22–24 October	Beaufort Sea: Herschel Island	Newport
Seal	12	1894	7–8 November	Beaufort Sea: Herschel Island	Newport
Duck	69	1895	2 October	Beaufort Sea: Herschel Island	Beluga
Rabbit	178	1895	12 February	Beaufort Sea: Herschel Island	Newport
Fox	30	1895	21 February–17 April	Beaufort Sea: Herschel Island	Newport
Duck	21	1895	9–21 October	Beaufort Sea: Herschel Island	Newport
Deer	46	1895–1896	17 December–21 January	Beaufort Sea: Herschel Island	Newport
Rabbit	39	1896	21 January, 7 March	Beaufort Sea: Herschel Island	Newport
Deer	23	1896	23 March–21 May	Beaufort Sea: Herschel Island	Newport
Duck	21	1896	26 May–21 June	Beaufort Sea: Herschel Island	Newport
Duck	152	1897	6–29 September	Beaufort Sea: Langton Bay	Beluga
Grouse	16	1897	8 September	Beaufort Sea: Langton Bay	Beluga
Seal	13	1897	6 September–12 December	Beaufort Sea: Langton Bay	Beluga
Duck	25	1897	23–24 September	Beaufort Sea: N. Alaska Coast	Navarch
Grouse	17	1897	8 September	Beaufort Sea: Langton Bay	Beluga
Deer	20	1897–1898	7 September–6 June	Beaufort Sea: Langton Bay	Beluga
Duck	164	1897–1898	6 September–27 June	Beaufort Sea: Langton Bay	Beluga
Seal	11	1897–1898	6 September –12 June	Beaufort Sea: Langton Bay	Beluga
Ptarmigan	39	1898	9 February–23 April	Beaufort Sea: Langton Bay	Beluga
Duck	34	1898	16–22 July	Beaufort Sea: Cape Bathurst	Beluga
Duck	16	1900	23 June	Bering Sea: Cape of Prince Wales	Beluga

**Table 4:** Diversity of catch over time. The species richness and the Shannon index of diversity (H) for all nonincidental harvest (>10 individuals of one species taken by a single vessel) by decade. Note that the first and the last decade each represent <10 years of data

Decade	Species richness	Shannon Index of diversity (H)
1850s	3	1.01
1860s	1	n/a
1870s	1	n/a
1880s	4	0.9
1890s	8	1.51
1900s	3	0.98

**Table 5:** Estimates of annual take by whalers on Hershel Island in the 1890s. Estimates are based on reported catch by the steam bark, Newport, over three seasons (1893–1896; Table 1). We assumed an average crew size of 36 individuals (M. Dyer pers. com) and that other whalers on Hershel Island were hunting in a similar manner. The range of estimated annual take values includes extrapolation of reported catches as both mean and median values

Species	Estimated annual take on Hershel Island
Rabbit	3,014–4,521
Deer	1,917–2,014
Ptarmigan	1,653–4,958
Eider duck	1,333–4,000
Duck	875–1,847
Fox	417–1,250
Seal	167–500

Within this limited timeframe, there was additional evidence for a collecting pattern with several examples of large numbers of animals being collected over a short period of time due to shifting resource exploitation patterns. For example, the number of walruses captured rose 500‐fold between the 1850s and 1860s and then collapsed. In addition to this sustained catch, there were also episodes of brief and intense catches in other taxa, for example, 521 of 524 cod (99.4%) were caught on 3 days in 1889, and 178 of 949 (18.3%) ducks were collected in September 1897. Thus, the spatial and temporal aspects of the harvest were varied by taxa, as were the subsequent ecological impacts.

Due to the preponderance of walruses in the reported catch virtually, all of the recorded catch were caught for both food and commercial good. Only 2.9% of species recorded were caught primarily for food (Table 2). Similarly, the numbers of walruses in the data resulted in the vast majority of biomass (~95%) recorded being from semiaquatic animals (Table 2).

## 4 Discussion

**Figure 2:**
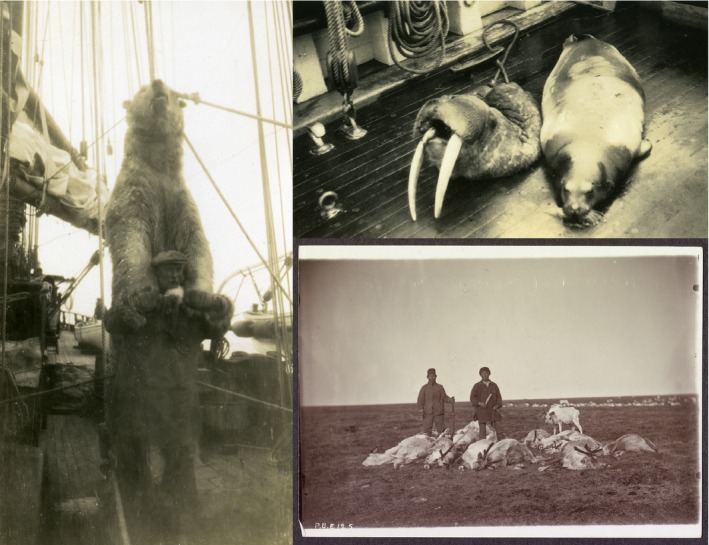
Examples of nonwhale animals targeted by the 19th Century American Whaling Fleet. Clockwise from top walrus and fur seal (New Bedford Whaling Museum (NBWM1988.6.3), caribou (NBWM 2000.100.200.33 “The Last of the Slaughtered Deer”), and Polar Bear (NBWM1988.6.11 “Polar bear off Wrangel Island”

The collateral damage of the large whale hunts of 19th Century American whaling vessels was taxonomically broad, while the majority of nongreat whale biomass came from a single economically important group—walruses which supports our first hypothesis (recoded catches would have an emphasis on economically valuable species.). However, a closer examination of the catches show that the species targeted included a large diversity of other species including terrestrial organisms. The diversity of organisms captured reflects the realities of maintaining a ship's crew and economic bottom line over multiyear voyages. As expected, there are a large number of marine species, including a variety of cetaceans and other marine mammals, turtles, and fish (Figure 2). While the walrus data were not surprising (Bockstoce & Botkin, 1982), what was unanticipated, was the diversity of terrestrial animals that were also captured by these ostensibly marine voyages.

Many of the terrestrial species were taken in northern latitudes (Table S1), while vessels were searching for more sought after whale species. For example, the bowhead whale, *Balaena mysticetus*, is a cold‐water specialist and was highly prized by Yankee whalers (Smith et al., 2012). The seasonal migrations of the animals coincided with the increasing daylight and subsequent increase in primary productivity in Arctic waters (Braham, Fraker, & Krogman, 1980). Whalers arriving ahead of these migrations would heighten their capacity to capture the greatest number of whales. Thus, it was not uncommon for ships to arrive early and prolong their stay, to maximize exploitation of the resource. Due to the vagaries of northern storms, ships were occasionally trapped in sea ice. For example, in September 1871, 40 American ships were frozen in the ice off of Port Franklin, Alaska. Thirty‐two of 40 ships (including the *Henry Taber,* the *Navy* the *Seneca*, and the *John Wells* whose logs we included in this study) were crushed in ice and lost (Starbuck, 1878).

During the times when the vessels were close to shore (or trapped in ice), away teams were sent out to provision the vessels. This provided American whalers the opportunity to capture terrestrial and coastal animals such as ducks, ptarmigan, fox, deer, bear, moose, and, at least on one occasion, two kangaroos. Sailors in the high Arctic targeted caribou, as they believed the meat could counteract scurvy (Hadley, 1915). While the local impacts on the local ecology could be severe (see discussion of Hershel Island below), it is unlikely that whalers captured enough individuals to have a substantive impact across the entire range (Table 2).

The temporal analysis reveals that much of this exploitation occurred in a heterogeneous fashion, in conjunction with our second hypothesis—that technology and exploitation patterns will lead to shifts in the places and kinds of species targeted. In our data, there is a clear trend to an increase in nongreat whale catch post civil war and that reflects improvements in vessel design, such as the transition from sail to steam as the major form of propulsion, as well as the introduction of Civil War veterans who were well trained in using fire arms. Coupled with the need for provisions (above), these factors lead to incidences of brief, localized, yet intense exploitation. For example, from 24 March through 9 April 1891, 170 individual grouse were captured, while 521 individual cod were caught over a 3‐day period (13–15 April 1889). These catch records demonstrate the sporadic and opportunistic nature of the opportunistic catch, with the harvest being characterized as having a high variance, with multiple days of inactivity punctuated by a few rare but high intensity harvesting events mediated by both the movements of the fishery and the limited opportunities for capture of targets.

In addition to the need for provisioning, falling whale oil prices lead to the need to target species that could be of secondary commercial importance. The walrus boom of the mid‐ to late‐1800s resulted in the taking of upwards of 235,000 walruses by the American fleet with 90% of that occurring between 1867 and 1883 (Table S1, Bockstoce & Botkin, 1982), a total that represents the approximate modern census size of all walrus populations (Lowry, Kovacs, & Burkanov, 2008). Our data show 2,283 individual walruses being captured. Based on the 60%–70% capture efficiency presented in Bockstoce and Botkin (1982), the whalers in our data set killed a minimum of 3,192 walruses. Several forces led to the start of this walrus boom. Access to walruses was improved after The United States purchased Alaska in 1867, obtaining legal claim over the walrus populations therein. This period also coincided with reductions in bowhead whale populations and a steady market for walrus products (Bockstoce & Botkin, 1982). Walruses therefore temporarily offered monetary compensation for lost bowhead products. The period lasted approximately 20 years during which contemporary researchers and naturalists began to recognize how hunting by whalers posed a conservation threat to walruses and to the Indigenous communities that depended on them. Reports from the time indicate that as early as the 1880s, the walrus population had been reduced by at least 50%; Nelson et al. (1887) report: “*it is only a matter of a few years when they (*the walrus) *will become comparatively rare where formerly abundant, and unknown in many of their former localities.”* (p. 270). These early years of commercial hunting only portended additional cycles of overexploitation and recovery of walrus stocks (Fay et al., 1989).

### 4.1 Data limitations

One of the major limitations to this study, and indeed many historical ecology studies in general, is that modern researchers are restricted to the quality of the data within the historical record (Josephson, Smith, & Reeves, 2008; McClenachan et al., 2015). In this paper, this limitation has three manifestations. One of these is recording bias: We can only tell what was captured when it was written down. Commonly captured organisms such as tuna or groupers may not have been mentioned, and each log is subject to the idiosyncratic threshold of what the author decided was worth mentioning. This introduces biases both within and between logs, and therefore, the numbers and categories we present here should be viewed as absolute minima. Our data contain an internal control illustrating this point. We have two logs (KWM 370 and ODHS 848) that were both kept aboard the *Betsy Williams* during her voyage from 1851 to 1854. In one log (KWM 370), the author recorded catching two sunfish, the second log (ODHS 848) recorded catching 23 porpoises, three turtles, one cod, one grouper, one skipjack, and the aforementioned sunfish. This example highlights how the recorded data should represent *an absolute minimum* estimate.

The second limitation centers on locality information. Often, the exact location of where the species were targeted was often not recorded. While we are able to record information at the scale of ocean regions or basin, more spatially explicit information was only recorded for a limited number of records (Table S1) and therefore we are unable to make more detailed analysis as to the spatiotemporal patterns of species capture.

The third limitation lies in trying to navigate the targeted species’ taxonomy. The people recording the logs were not trained scientists, and while they had intimate knowledge of the behavior and ecology of the large whales, they were unencumbered with formalized spelling rules, consistent common names, or widely accepted taxonomy (Townsend, 1925). For example, the animal to which whalers referred to as “grampus” is unclear, and the term may have applied to a number of cetacean species. Overall, it appears that grampus may have been a very general word used to describe many species of dolphins (Family Delphinidae) and beaked whales (Family Ziphiidae) (M. Dyer, personal communication) and we have chosen the (relatively) common Cuvier's Beaked Whale (*Ziphius cavirostrus*), for our biomass calculations.

### 4.2 Conservation implications

Conservation of future populations requires understanding of historical antecedents (Thurstan et al., 2015). Characterizing past conditions allows us to differentiate between anthropogenic and climate driven cycles in abundance (Schwerdtner Máñez et al., 2014), to model ecosystem productivity (Rosenberg et al., 2005) and to reconcile past species distributions (Drew, Philipp, & Westneat, 2013). While we urge caution when dealing with conclusions drawn from incomplete historical data, in many cases these data represent the only insight we have into the less perturbed past of ecosystems (Hayashi, 2014; Schwerdtner Máñez et al., 2014). Ignoring these data runs the risk of setting the conservation bar too low.

**Figure 3:**
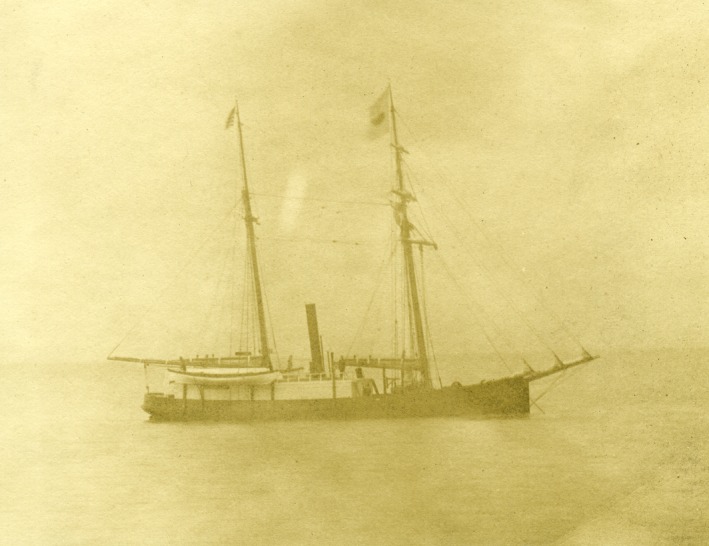
The Mary B. Hume off of Herschel Island (NBWM 1988.6.195) Vessels like the one pictured here overwintered in Arctic waters to capture bowhead Whales. While waiting for the ice to melt, they sent hunting and trading parties onto the land with ecological and social impacts to the animals and people living in those areas

Our results provide critical insight into what past coastal ecosystems, particularly boreal regions, must have looked like in the 19th century. Moreover, they speak to how historical human resource exploitation may influence modern ecological studies. While the range‐wide impacts across a population may have been minimal for terrestrial organisms, the episodic and spatially localized nature of whalers’ harvests could mean that these marine voyages had demonstrable impacts on specific and localized terrestrial communities. For example, Herschel Island in the Beaufort Sea has been the focus of several recent ecological studies (Burn & Zhang, 2009; Dickson & Gilchrist, 2002; Kokelj, Smith, & Burn, 2002; Lantuit & Pollard, 2008; Myers‐Smith et al., 2011) focusing on the climate change and land cover. During the 19th century, Herschel Island was the largest whaling settlement of this region and was the site for vessels pursuing bowhead whales (Fraker & Bockstoce, 1980; Figure 3). During the 1890s, the estimated population size of 1,500 people (Bockstoce, 1986). Our limited sampling of the total whaler efforts showed that crews of vessels captured 316 ducks, 158 “deer” (most likely caribou), 36 foxes, 11 grouse, 120 ptarmigan, 149 rabbits, 21 seals, and one bear from Herschel Island. Similarly, Bockstoce (1980) suggested whalers took over 12,000 caribou from Herschel Island between the periods 1890 and 1908. Modern studies looking at how the ecosystem including the community ecology and nutrient cycling patterns of the region have changed over time needs to factor in the magnitude of biomass removal. Only by doing this will researchers be able to set adequate targets for restoration and conservation.

In contrast to localized terrestrial impacts, walruses faced massive declines across their ranges due to unregulated hunting from both opportunistic whalers and targeted walrus hunts. The harvest data indicate that current walruses have gone through at least three anthropogenic population declines (Fay et al., 1989) although these bottlenecks may have occurred too recently to be reflected in molecular analyses (Andersen et al., 2009). Modern distribution of walruses, and the associated high levels of population connectivity, may be a result of population expansion into areas that were defaunated by whalers (Wiig, Gjertz, & Griffiths, 1996).

Additionally, the impacts of the whaling and walrus hunting on the Indigenous cultures that were dependant on those species were not overlooked by contemporary authors. For example, Aldrich (1889) recounted that “*Whaleman have practically driven the walruses from the shore, and greatly reduced the numbers of hair seals and whales*. *Thus, all the supplies of food have been curtailed*.” The loss of both the bowhead whale and the reduction in walrus populations had negative consequences on the Indigenous tribes, resulting in loss of food, shifts in harvesting and migration patterns and urbanization around trading centers such as the one established in Herschel Island (Foote, 1964; Hadley, 1915). The rapid transition of Herschel Island into a whaling center had at least two impacts on the Indigenous population. First, it changed their annual trading voyages and leads to a centralization of the population. With the establishment of a trading outpost on the island, the population had less reason to migrate, especially because the store offered processed food. The importance of this store was reflected in the native language with the word *iglupûk* meaning big house, or in the context of Herschel Island, the Hudson Bay Trading company (or on occasion, the police barracks—Stefansson, 1909; ). Second, the sailors would also commission the Indigenous people to hunt caribou, fish, and ptarmigan, often paying for those goods in flour, molasses, and canned meats (Hadley, 1915). This shift in dietary preferences portended current concerns of cardiometabolic health among Indigenous peoples of the high Arctic. (Ryman et al., 2015).

Our data show that Yankee whalers targeted a number of species, both marine and terrestrial during their search for whales. We also show the number of these nongreat whale targets changed over both time and space, and while locally intense, the take of terrestrial organisms was probably insufficient to cause range‐wide declines in terrestrial animals. However, we did show that there were substantial impacts to commercially valuable semiaquatic organisms such as walruses, with impacts on both biological and cultural diversity in the far north. Our work shows that Yankee whalers had a wide‐ranging impact on marine ecosystems in general but also on localized terrestrial ecosystems. Logbooks of 74 vessels covering 79 voyages contain a sample of the vivid splendor of past ocean ecosystems. When one extrapolates the take of nontarget species from our small sample of 79 voyages out to the entirety of the American Fleet, estimated at over 1,600 voyages (Townsend, 1935), it becomes clear that commercial whalers represented a nontrivial removal of nonlarge whale biomass from terrestrial and marine systems.

## Conflict of Interest

None declared.
